# Cardiac Murmur in a Boy with Normal Paternal Prenatal Carrier Screening for Pompe Disease

**DOI:** 10.1155/2019/6274979

**Published:** 2019-12-12

**Authors:** Allison M. Jay, Premchand Anne, David Stockton

**Affiliations:** ^1^Division of Genetics, Ascension St. John Hospital, Detroit, MI, USA; ^2^Division of Pediatric Cardiology, Department of Pediatrics, Ascension St. John Children's Hospital, Detroit, MI, USA; ^3^Department of Genetics, Children's Hospital of Michigan, Detroit, MI, USA

## Abstract

**Introduction:**

Pompe disease is an autosomal recessive lysosomal storage disorder with marked morbidity and mortality, if untreated. With the advent of enzyme replacement therapy, it is essential to identify the infantile-type as early as possible to mitigate the effects of the enzyme deficiency. Identification is possible prenatally with testing of both parents. More recently, many states have instituted newborn screening for this condition.

**Case:**

We report a patient with infantile-onset Pompe disease with a normal paternal carrier genetic test, born prior to newborn screening for Pompe disease in the state of Michigan. The infant's father was retested once the infant was diagnosed with Pompe disease postnatally and noted to have a mutation conducive to Pompe disease.

**Conclusion:**

Providers should have a strong clinical suspicion for disorders even if prenatal parental carrier screening is normal. A normal parental prenatal test does not exclude the possibility that the fetus may be diagnosed with a disorder postnatally, and pediatricians may be faced with limitations in accuracy of parents' recollection of parental testing results.

## 1. Introduction

Pompe disease (OMIM 232300; glycogen storage type II; *α*-glucosidase deficiency) is an autosomal recessively inherited lysosomal storage disorder which occurs in approximately 1 in 40,000 infants. Infants typically present as “the floppy infant” with hypertrophic cardiomyopathy [1]. These infants with the classic type had a high mortality with death within the first year of life [2]. A later-onset form presents in childhood with skeletal muscle dysfunction and calf hypertrophy mirroring Duchene muscular dystrophy. Since 2006, enzyme replacement therapies have reduced the mortality and morbidity of this condition. Typically, patients will receive intravenous (IV) infusions every few weeks and have improved skeletal and cardiac function. Given a treatment that improved clinical outcomes, Pompe disease was added to the US Secretary of Health and Human Services on the Recommended Uniform Screening Panel in 2015 [3]. Through a blood spot sent in commonly in the newborn period, infants can now be detected if their state offers Pompe screening. Here we report an infant diagnosed with Pompe disease who was born two weeks prior to initiation of newborn screening (NBS) in the state of Michigan, who presented to his pediatrician at 4.5 months with a cardiac murmur. His parents had undergone an extensive prenatal screen and did not think Pompe disease was a likely diagnosis given the father's reported normal result.

## 2. Case Report

**Figure 1:**
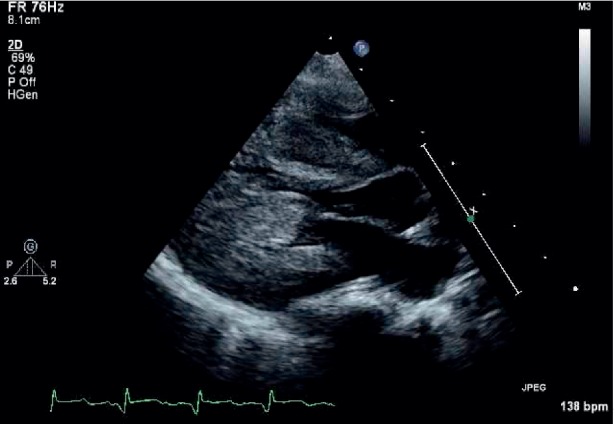
Parasternal long axis view of the patient's left ventricle showing marked concentric hypertrophy.

A 4.5-month-old infant presented to his pediatrician for his 4-month well-child check. He was noted to have a 3/6 harsh, systolic ejection murmur along the left sternal border without radiation. He was subsequently referred for consultation to pediatric cardiology at our community hospital. The echo cardiogram (Figure 1) showed marked hypertrophy of the ventricle, and the electrocardiogram (ECG) indicated marked biventricular hypertrophy by voltage criteria. The infant also had mild, diffuse hypotonia. With the concern for Pompe disease, an *α*-glucosidase enzyme level was drawn. He was subsequently admitted to our Pediatric Intensive Care Unit to monitor him during initiation of propranolol for the marked hypertrophy. While in the PICU, the genetics service was consulted to evaluate for Pompe disease.

Upon gathering prenatal history and family history for a genetic evaluation, the mother reported having fertility treatment, and extensive prenatal genetic testing that both she and the father of the baby had undertaken. The mother spoke to a genetic counselor on the phone regarding her results which were positive for a mutation in acid *α*-glucosidase (*GAA)* gene associated with Pompe disease. She provided a copy of the results which confirmed the mutation in *GAA*, c.784G>A(p.Glu262Lys). The father's results were reported by the same company and were negative; a report for the father's results was provided by the family. The screening panel for both had included 120 diseases, and the metabolic disorders screened are outlined in Table 1.

Family history showed maternal ancestry of being Sicilian and Polish, and paternal ancestry was significant for Scottish and English. There were no children with birth defects, no individuals with cardiac problems, and there was no known consanguinity. The infant was the first child of his parents. Physical examination showed a weight of 8.12 kg (>90%) and was remarkable for macroglossia, generalized hypotonia, and head lag.

The *α*-glucosidase activity level was reported to be low at 0.7 nmol/hr/mg (ref 5.5–25). The patient was transferred to an academic institution where molecular testing of the parents was repeated, and the patient was initiated on enzyme therapy. Parental testing confirmed the mother's mutation that was previously reported and identified the father as having a mutation in the *GAA* gene: c.1082C>T(p.Pro361Leu) and characterized as causative of disease. The family later provided an amended report from the prenatal company confirming the c.1082C>T *GAA* mutation by Sanger sequencing.

After initiation of ERT, the patient has done well, and his cardiac findings have improved. The indexed left ventricular mass (LVMI) has been followed from the initial echocardiogram to the repeated echocardiograms; the initial LVMI was 121.8 g/m^2.7^, with the repeat LVMI of 64.1 g/m^2.7^ after two months of ERT. There was also marked reduction in left ventricular intracavitary gradient from 100 mmHg to 54 mmHg. The LVMI continued to decrease to normal levels, along with the resolution of the intracardiac gradient and the resultant murmur on physical exam. He is continuing to receive 20 mg/kg infusion of alglucosidase alfa weekly. He continues to remain CRIM-negative.

## 3. Discussion

Parental carrier testing in the prenatal setting is more expansive than ever, with many metabolic disorders screened. This patient's case highlights that a normal result on a parental carrier screen does not exclude a diagnosis in a child. Patients should be counseled about the limitations of genetic technology, and understandably with the amount of conditions tested for they may not recall whether they were positive or negative for a specific condition. Pediatricians should also be aware of the limitations of current genetic technology; while in the past Sanger sequencing was used today, many current panels use next generation sequencing (NGS) for patient testing [4]. This method allows for the generation of massive amounts of data from an individual patient through sequencing millions of fragments in a parallel manner. Pediatricians should be aware that read depth and coverage, which indicate how well a region of the gene is interrogated, may vary from lab to lab and may be a source of error in patient testing if this is inadequate. Coverage of GC rich regions and areas of high homology to other regions in the genome, for instance, is always challenging. In addition, some laboratories only offer targeted testing of certain mutations, so while a negative result may indicate the absence of any of the targeted mutations, this does not exclude the possibility entirely of another mutation elsewhere in the gene. Finally, there is the possibility of gonadal mosaicism in which a parental carrier screen on peripheral blood would not identify a mutation.

Pediatricians are often on the front lines taking histories and gathering information on prenatal history and may be faced with limited recollections as well as with parents who bring extensive paperwork with their prenatal carrier screens. Pediatricians may be asked to interpret these parental reports and apply them to the child before them. It is prudent to look at positive findings from such reports and take that into the clinical differential if a patient is having symptoms, and also equally important not dismiss a potential diagnosis based on a parent's recollection their screening was normal. While our patient received genetic counseling over the phone, the patient's parents still believed that Pompe could not be the cause of their child's condition given a negative result on the father's prenatal screen.

In conclusion, the symptoms of hypotonia, a cardiac murmur, and hypertrophic cardiomyopathy with marked intracavitary obstruction suggested the diagnosis of Pompe disease, which was subsequently confirmed by genetic testing. Astute clinical judgement is needed in cases where molecular testing is inconsistent and strong clinical suspicion may be needed for the infant presenting with cardiac and noncardiac signs and symptoms. There may be limitations with the genetic technology a patient has had performed which cause a mutation to be missed as has been reported in one case with Pompe by Tsai et al. [5]. In such cases, biochemical testing may be warranted to further evaluate these patients through checking enzymatic activity. With the addition of Pompe disease to the newborn screening in many states, this will allow for the earlier detection of this condition; however, there are numerous other biochemical disorders that may affect patients presenting to pediatrician. It is prudent to review the prenatal results and evaluate the need for additional workup if patients' symptoms are concerning for a metabolic condition. Parents may not recall what their parental carrier testing results were accurately; it is important for the physician to confirm the results with an official report, and to be cognizant that a normal result does not exclude a diagnosis.

## Figures and Tables

**Table 1:** Genetic/metabolic disorders tested for on parental prenatal carrier panels.

3-Phosphoglycerate dehydrogenase deficiency	Hereditary thymine-uraciluria
Abetalipoproteinemia	Homocystinuria
Alkaptonuria	Hurler syndrome (mucopolysaccharidosis type 1)
Alpha-mannosidosis	Hypophosphatasia
Aspartylglucosaminuria	Isovaleric acidemia
Biotinidase deficiency	Krabbe disease
Canavan disease	Long chain 3-hydroxyacyl-CoA dehydrogenase deficiency
Carnitine palmitoyltransferase IA deficiency	Maple syrup urine disease
Carnitine palmitoyltransferase II deficiency	Medium chain acyl-CoA dehydrogenase deficiency
Classic citrullinemia	Metachromatic leukodystrophy
CLN3-related neuronal ceroid lipofuscinosis	Mucolipidosis IV
CLN5-related neuronal ceroid lipofuscinosis	Multiple sulfatase deficiency
Congenital disorder of glycosylation, type Ia	Niemann-Pick disease type C
Congenital disorder of glycosylation, type Ib	PEX1-related Zellweger syndrome spectrum
Cystinosis	Phenylalanine hydroxylase deficiency
D-bifunctional protein deficiency	Pompe disease
Dihydrolipoamide dehydrogenase deficiency	PPT1-related neuronal ceroid lipofuscinosis
Galactosemia	Primary carnitine deficiency
Gaucher disease	Primary hyperoxaluria type 1
Glutaric acidemia type Ia	Primary hyperoxaluria type 2
Glycogen storage disease type 1a	Pycnodysostosis
Glycogen storage disease type 3	Rhizomelic chondrodysplasia punctata type 1
Glycogen storage disease type V	Salla disease
Hereditary fructose intolerance	Segawa syndrome
Tay-Sachs disease	Short chain acyl-CoA dehydrogenase deficiency
TPP1-related neuronal ceroid lipofuscinosis	Smith-Lemli-Opitz syndrome
Type I tyrosinemia	Wilson disease
